# The Bonn-UFO technique: A rapid and reproducible approach for aortic root and intervalvular fibrous body reconstruction

**DOI:** 10.1016/j.xjtc.2026.102503

**Published:** 2026-07-08

**Authors:** Ali El-Sayed Ahmad, Saad Salamate, Ali Bayram, Kaveh Eghbalzadeh, Marwan Hamiko, Farhad Bakhtiary

**Affiliations:** Department of Cardiac Surgery, University Hospital Bonn, Bonn, Germany

**Figure:**
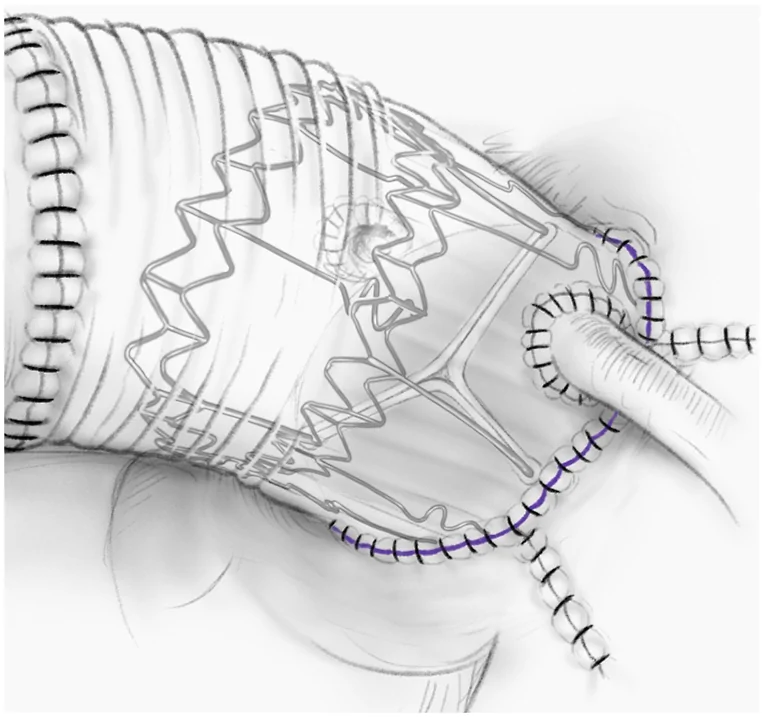
Sutureless aortic valve and direct coronary reimplantation are used in the Bonn-UFO technique.

**Central Message**

Bonn-UFO technique streamlines the UFO operation with an inverted-everted Valsalva graft, direct coronary reimplantation, and a sutureless valve, thereby reducing anastomoses and potentially shortening ischemic times.

Surgical treatment of complex endocarditis affecting the aortic and mitral valves, with destruction of the aortic root (AR) and the intervalvular fibrous body (IVFB), requires time-consuming, extensive, and complex reconstruction techniques to effectively restore cardiac anatomy and function.^1^^,^^2^ We previously described our technique for reconstructing the AR, the left ventricular outflow tract (LVOT), and the IVFB with neighboring structures after radical resection for extensive infective endocarditis.^1^^,^^2^ Although clinically effective, these procedures are associated with increased perioperative morbidity and mortality.^3^^,^^4^ However, the complexity of the procedure remains a significant limitation of traditional approaches, prompting further refinements. To address these limitations, we developed the Bonn-UFO technique (B-UFO). Some technical elements of B-UFO, particularly the use of an inverted and everted Valsalva graft and a sutureless valve, are based on our recently described modified Bentall procedure.^5^ In the present work, these principles are adapted to the distinct and more complex setting of UFO reconstruction after radical debridement involving the AR, the LVOT, the IVFB, and the mitral-aortic continuity. The technique uses an inverted and everted Valsalva graft secured with a single continuous suture, direct coronary button reimplantation, and rapid deployment of a sutureless self-expanding aortic prosthesis. The current article describes this novel surgical strategy, detailing its technical refinements and advantages over the classical UFO procedure.

## Operative Technique

### Shared Steps With the Classic UFO Procedure

The classical UFO technique has been described in detail.^1^^,^^2^ Briefly, after median sternotomy, cardiopulmonary bypass (CPB) is established via bicaval cannulation, the ascending aorta is crossclamped, and cardioplegia is administered. An oblique aortotomy is made toward the noncoronary sinus and is subsequently connected to an incision in the left atrial roof, sometimes extending across the interatrial septum into the right atrium, thus exposing both valves and the IVFB. Radical debridement is performed to remove the infected, calcified, or compromised tissues, including the anterior mitral leaflet and the destroyed IVFB, resulting in a single aperture connecting the left ventricular inflow and outflow tracts. Mitral valve replacement is performed first, using pledgeted 2-0 polyester sutures placed from the posterolateral to the anteromedial trigone along the posterior annulus. A prosthetic mitral valve is implanted, carefully positioned to prevent LVOT obstruction.

A folded double-layer patch of bovine pericardium reconstructs the IVFB and left atrial roof. The closed edge is sutured to the anterior mitral sewing cuff, thereby reconstructing the IVFB while and closing the atrial roof incision posteriorly. The anterior portion of the patch remains available for integration into the subsequent AR reconstruction. Both trigonal junctions are reinforced for complete hemostasis and stability.

### Bonn-UFO Technique

A Vascutek Valsalva graft (Gelweave Valsalva; Vascutek Terumo Inc.) is trimmed and inverted into the LVOT. The proximal skirt is secured with a continuous 4-0 polypropylene suture placed circumferentially around the aortic annulus, first in a clockwise direction across the right and noncoronary cusps, as well as the anterior portion of the bovine pericardium patch, and then in a counterclockwise direction around the left coronary cusp, thereby achieving a robust hemostatic seal (Figure 1). The graft is then everted back to its normal configuration, thereby forming a seamless conduit from the LVOT to the ascending aorta (Figure 2). In our experience, appropriate trimming of the proximal skirt and the use of a single row of continuous sutures create a smooth neoannular collar after eversion rather than a bulky inward fold, and this adjustment does not hinder annular sizing or subsequent valve deployment.

**Figure 1:**
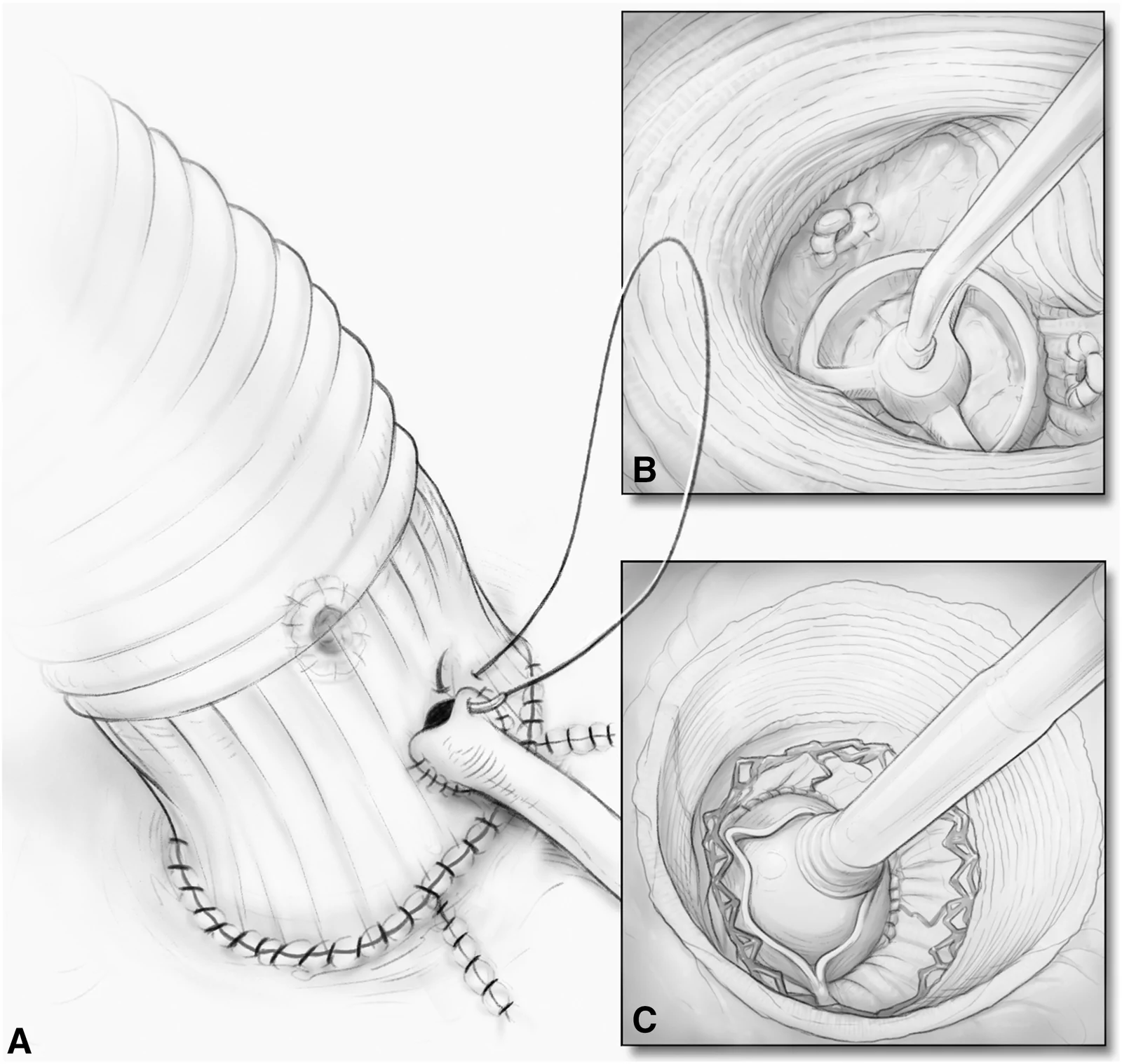
Bonn-UFO technique valve implantation sequence. A, Left coronary button anastomosis. B, Annular sizing after coronary implantation. C, Valve commissural alignment postdeployment.

**Figure 2:**
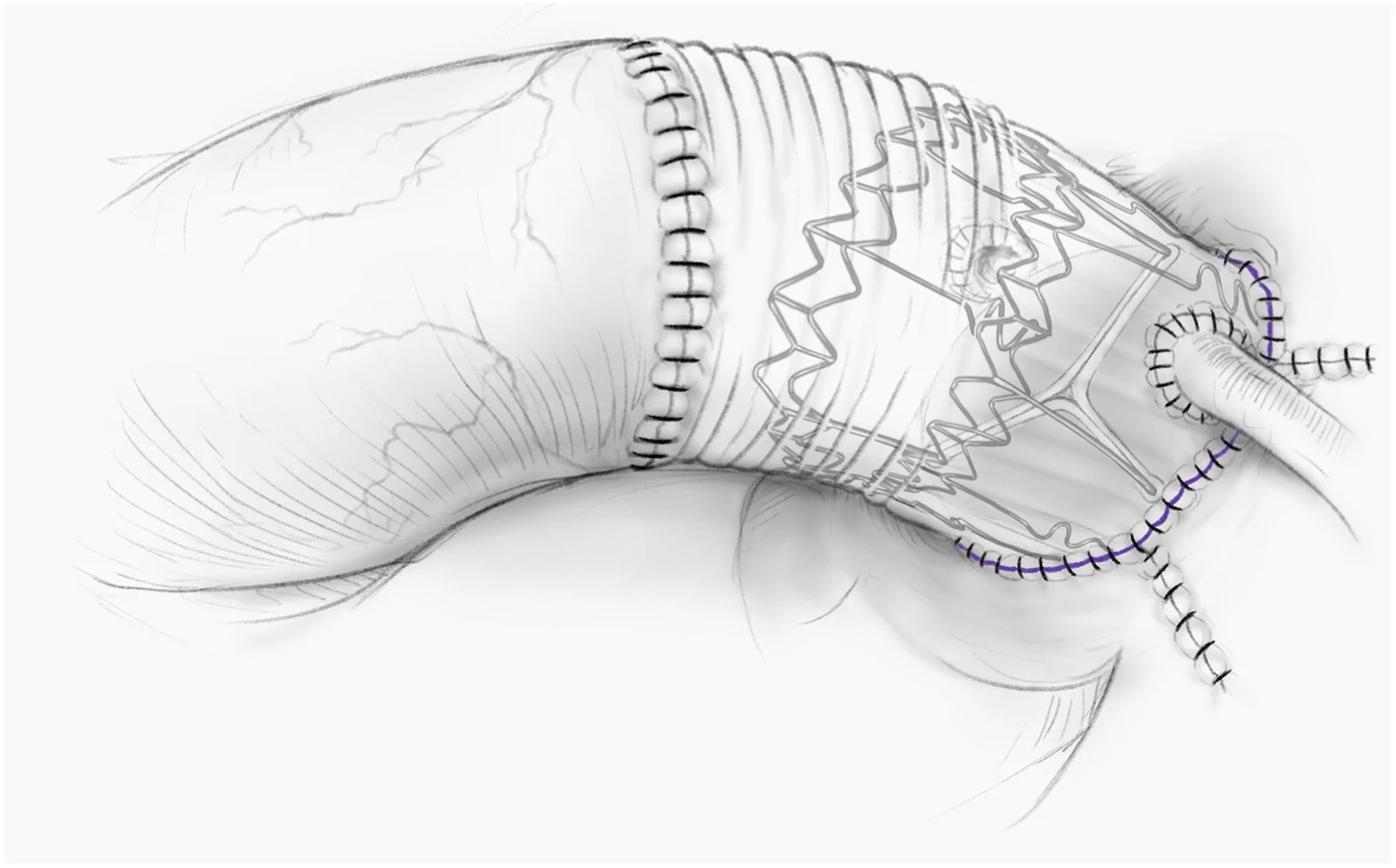
Final Bonn-UFO technique reconstruction illustrating Valsalva graft, continuous annular suture, self-expanding valve, and direct coronary button implantation.

Coronary reimplantation is performed immediately. Well-defined neo-ostia are created in the Valsalva graft, and the coronary buttons are directly sutured into these openings using continuous 4-0 polypropylene sutures, thereby eliminating the need for interposition grafts (Figure 2).

Subsequently, a self-expanding sutureless valve is selected based on intraoperative annular sizing (Figure 1, *upper panel*) and is deployed inside the conduit. Careful rotation ensures proper commissural alignment with the coronary ostia for unobstructed coronary flow (Figure 1, *lower panel*).

Finally, the distal Valsalva graft is anastomosed to the native ascending aorta using a running 3-0 polypropylene suture. Routine deairing, echocardiographic verification, and standard weaning from CPB finalize the procedure. A surgical video demonstrating the specific steps of the B-UFO, performed in a patient undergoing this operation, is provided (Video 1).

## Comments

The B-UFO streamlines the original UFO procedure by introducing 2 pivotal changes: direct implantation of coronary buttons into the Valsalva graft and the use of a self-expanding, sutureless aortic valve. Although these technical elements overlap with our recently modified Bentall approach,^5^ the present procedure applies them to a different and more complex reconstructive problem after radical debridement of destructive endocarditis involving the AR, the LVOT, and the IVFB. Together, these steps can avoid up to 3 anastomoses: the 2 Cabrol-type coronary extension grafts, by directly sewing the coronary buttons into the graft, and the complete circumferential valve-to-graft sewing step. This reduction in suture lines could significantly reduce myocardial ischemia and CPB durations, an efficiency that is important given the well-documented association between longer crossclamp/CPB times and worse outcomes,^3^^,^^4^ while also providing fewer potential bleeding sites.

The advantage of the sutureless prosthesis extends beyond minimal time savings. By sparing a complete circumferential valve-sewing step within a deeply reconstructed field, it reduces proximal suture burden and may facilitate hemostasis. Comparative studies do not isolate annular sewing time specifically, but sutureless valves consistently shorten operative times compared with conventional sutured prostheses, with reported reductions of 15 to 17 minutes in crossclamp and/or CPB times.^E1^^,^^E2^ In addition, deployment following proximal conduit fixation allows low-position valve implantation within the graft and adjustment of the spatial relationship between the valve frame, graft sinuses, and coronary ostia, which may facilitate direct coronary reimplantation without extensions. For the same reason, a prefabricated composite graft would not fully replicate the present strategy because it would forfeit the flexibility of implanting the valve only after the conduit has been secured and the coronary geometry assessed. Meta-analyses further suggest that sutureless valves are associated with fewer transfusions and less postoperative renal dysfunction than conventional stented bioprostheses.^E3^

These principles may also be applicable to selected other complex root reconstructions, and sutureless valves may be attractive in settings such as a small aortic annulus.^5^^,^^E4^ However, our current experience is limited to UFO-type endocarditis reconstruction.

Early institutional experience is consistent with these expectations. Nine patients have undergone reconstruction with the B-UFO and experienced uneventful recoveries. No paravalvular leakage was observed. Median myocardial ischemia time was reduced by 20 minutes, to 58 minutes (interquartile range [IQR], 47.5-101), and median CPB time was 112 minutes (IQR, 80-185.5), compared with our historical classic-UFO cohort.

## Conclusions

The B-UFO maintains fundamental surgical principles while markedly simplifying double-valve, AR, and IVFB reconstruction. By eliminating complex anastomoses and integrating a sutureless valve with an inverted Valsalva conduit, the B-UFO reduces procedural complexity, shortens operative times, and holds promise for wider surgical application.

## Conflict of Interest Statement

F.B. reports relationships with Edwards Lifesciences, Medtronic, Corcym, and Abbott, which include consulting or advisory roles as well as speaking and lecture fees outside the submitted work. F.B. reports a relationship with LSI that includes speaking and lecture fees outside the submitted work. All other authors reported no conflicts of interest.

The *Journal* policy requires editors and reviewers to disclose conflicts of interest and to decline handling or reviewing manuscripts for which they may have a conflict of interest. The editors and reviewers of this article have no conflicts of interest.

## Supplementary Data

Video 1 Intraoperative demonstration of the Bonn-UFO technique in a single patient, highlighting the inverted-everted Valsalva graft, the single continuous annular suture, direct coronary button reimplantation, and deployment of a sutureless self-expanding valve. Video available at: https://www.jtcvs.org/article/S2666-2507(26)00310-X/fulltext.
